# Segregation of glutamatergic and cholinergic transmission at the mixed motoneuron Renshaw cell synapse

**DOI:** 10.1038/s41598-017-04266-8

**Published:** 2017-06-22

**Authors:** Boris Lamotte d’Incamps, Gardave S. Bhumbra, Joshua D. Foster, Marco Beato, Philippe Ascher

**Affiliations:** 1 Center for Neurophysics, Physiology and Pathologies, CNRS UMR 8119, Université Paris Descartes, Paris, France; 2 Department of Neuroscience, Physiology and Pharmacology, UCL, Gower Street, London, United Kingdom; 3 Physiologie cérébrale, CNRS UMR 8118, Université Paris Descartes, Paris, France

## Abstract

In neonatal mice motoneurons excite Renshaw cells by releasing both acetylcholine (ACh) and glutamate. These two neurotransmitters activate two types of nicotinic receptors (nAChRs) (﻿the homomeric α_7_ receptors and the heteromeric α*ß* receptors) as well as the two types of glutamate receptors (GluRs) (AMPARs and NMDARs). Using paired recordings, we confirm that a single motoneuron can release both transmitters on a single post-synaptic Renshaw cell. We then show that co-transmission is preserved in adult animals. Kinetic analysis of miniature EPSCs revealed quantal release of mixed events associating AMPARs and NMDARs, as well as α_7_ and α*ß* nAChRs, but no evidence was found for mEPSCs associating nAChRs with GluRs. Bayesian Quantal Analysis (BQA) of evoked EPSCs showed that the number of functional contacts on a single Renshaw cell is more than halved when the nicotinic receptors are blocked, confirming that the two neurotransmitters systems are segregated. Our observations can be explained if ACh and glutamate are released from common vesicles onto spatially segregated post-synaptic receptors clusters, but a pre-synaptic segregation of cholinergic and glutamatergic release sites is also possible.

## Introduction

The early view that chemical synaptic transmission is mediated by a single transmitter activating a single-receptor type has evolved since the observation that at some synapses a transmitter activates multiple classes of post-synaptic receptors, and that a given neuron can release multiple transmitters^1^. The motoneuron (MN) to Renshaw cell (RC) synapse is a typical example of this evolution: after the demonstration that the synapse involves nicotinic receptors (nAChRs)^2^ it was shown that acetylcholine (ACh) also activates muscarinic receptors^3^. It was later found that MNs can release both ACh and glutamate^4, 5^, and that ACh activates both homomeric (α_7_) and heteromeric (α*β*) nAChRs, while glutamate acts on AMPARs and NMDARs^6^. Thus at the MN-RC synapse two transmitters activate four different ionotropic receptor types. Understanding the functional interest of co-transmission and the respective roles of the four receptors will ultimately require establishing whether there is a pre-synaptic segregation of the two transmitters in different vesicles, and what is the distribution of the four receptors on the post-synaptic side. Here we try to address whether these four receptors are co-localized.

This question has been asked at other synapses bearing more than one ionotropic receptor to a given transmitter, or releasing more than one transmitter. If the two receptors are co-localized and have different kinetics, spontaneous quantal release will give rise to miniature post synaptic currents (mPSCs) with mixed kinetics, a feature that was used to demonstrate co-localization of AMPARs and NMDARs in cultured neurons^7–12^ and of nicotinic receptors containing α_3_ subunits and α_7_ nAChRs in the chicken ciliary ganglion^13, 14^. Similarly, mixed mPSCs are observed when two transmitters are released from the same quantum onto different post-synaptic receptors, as is the case for GABA and glycine^15–18^ for glutamate and GABA^19^ and for glutamate and ACh^20, 21^.

In the present study we first showed that co-transmission at the MN-RC synapse, which had until now only been observed in neonates, is not a transient developmental phenomenon, since we established its occurrence in preparations from adult mice. We then exploited the differences in kinetics among the nAChRs and GluRs and detected both mixed glutamatergic and mixed cholinergic mEPSCs, but never mEPSCs associating a nAChR and a GluR. This segregation was confirmed by quantal analysis of evoked EPSCs which showed a specific reduction in the number of functional release sites following pharmacological blockade of nAChRs.

## Results

### A single motoneuron can release ACh and glutamate, and co-transmission is present in both neonate and adult mice

**Figure 1:**
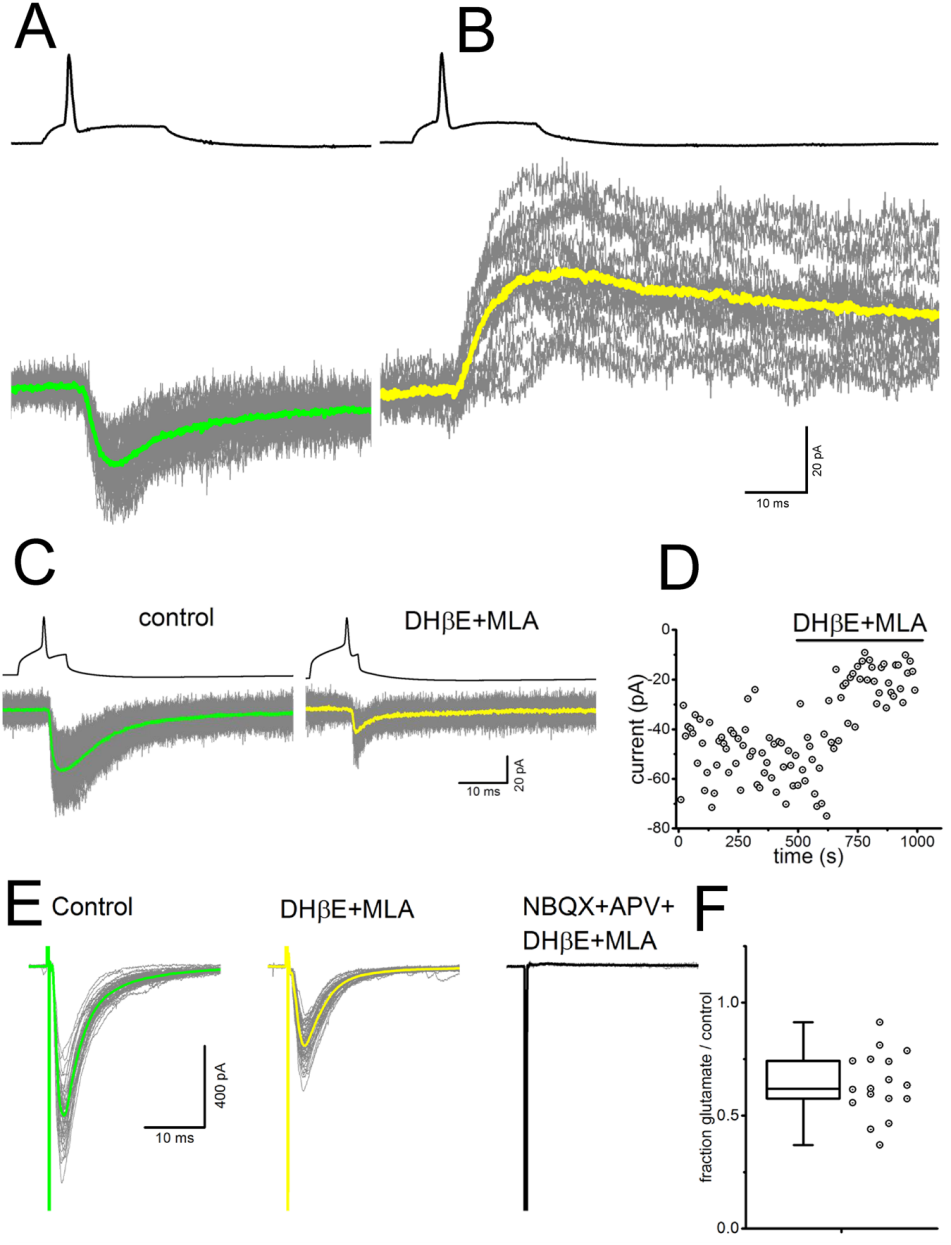
Glutamate and ACh co-transmission at young and adult MN-RC synapses. (**A–D**) Paired recording between a MN and a RC in slices from neonates (P5-P9). (**A**) In the presence of NBQX (2 µM) the action potential (single trace, top, after offline bridge correction) elicited by a square pulse of depolarizing current (not shown) in the MN, elicits an EPSC in the RC, recorded in voltage-clamp at a holding voltage of −60 mV, with a latency of 1.45 ms after the peak of the action potential. At negative potentials, most of the current is due to nicotinic receptors, since the NMDA component is affected by the Mg^2+^ block at negative potential. (**B**) At positive holding potential (+30 mV), the nAChRs mediated currents are blocked by their rectification properties and a slow NMDARs mediated current is observed (yellow trace). (**C**) Pharmacological evidence of co-transmission in another pair of RC-MN recorded in a young pup (P8): amplitude of the EPSC in the RC before and after the application of the nicotinic antagonists MLA (10 nM) and DHßE (10 µM). (**D**) Scatter plot of the time course of the amplitude of the EPSC in the RC before and during the application of the nicotinic antagonists. The RC is held at −60 mV. (**E**,**F**) Pharmacological evidence of co-transmission in adults. (**E**) The synaptic current elicited in a RC by ventral root stimulation is shown before (left, green averaged sweep), and during the application of nicotinic antagonists (middle, yellow averaged sweep). Application of glutamate receptors antagonists (NBQX 3 µM and D-APV 50 µM) abolishes the remaining synaptic current. (**F**) Fraction of the synaptic current that can be ascribed to glutamate receptors (amplitude of the current in DHßE + MLA/amplitude of the current in control conditions) in 17 RCs from adults (P21-P90).

We first confirmed the isolated observation of Nishimaru *et al*.^5^ who reported release of both glutamate and ACh from a single MN-RC pair. In three pairs of connected MNs and RCs we demonstrated co-transmission using the rectification properties of the two nicotinic currents, blocked when the cell is held at positive potentials, and those of the NMDARs, that are blocked by external Mg^2+^ ions when the RC is hyperpolarized^6^. After blocking AMPARs with NBQX (2 µM), pure nicotinic currents where observed when the cell was held at −60 mV (Fig. 1A). Holding the cell at +30 mV (Fig. 1B) abolished the nicotinic component and revealed a slow NMDA mediated current. This observation was confirmed by pharmacological blockade: in one connected pairs, application of blockers of both types of nicotinic receptors confirmed the presence of a synaptic (glutamatergic) current (Fig. 1C,D).

The results from paired recordings confirm that in juvenile animals a single MN can release both transmitters and activate both nAChRs and GluRs. Since co-transmission of GABA and glycine is known to be developmentally down-regulated^22^ we have performed a subset of experiments in fully mature animals (P21-P90) and measured the relative contribution of ACh and glutamate to the EPSCs evoked in RCs by ventral root stimulation. The response in control was reduced by the application of the two nAChRs antagonists DHβE and MLA, but was fully abolished only after additional application of glutamate antagonists (NBQX and APV, Fig. 1E). On average, glutamate contributed to 64 ± 14% (SD, n = 17, Fig. 1F) of the response, confirming that co-transmission is maintained in adult animals at this synapse.

Our paired recordings confirm the original observation by Nishimaru *et al*.^5^ that a single MN can release both transmitters, but neither indicate whether the two transmitters are stored in the same vesicle, nor whether post-synaptic receptors are co-localized. In order to address these issues we analysed the kinetics of mEPSCs in condition of isolation of one or two types of post-synaptic receptors at a time.

### Single-receptor type mEPSCs

**Figure 2:**
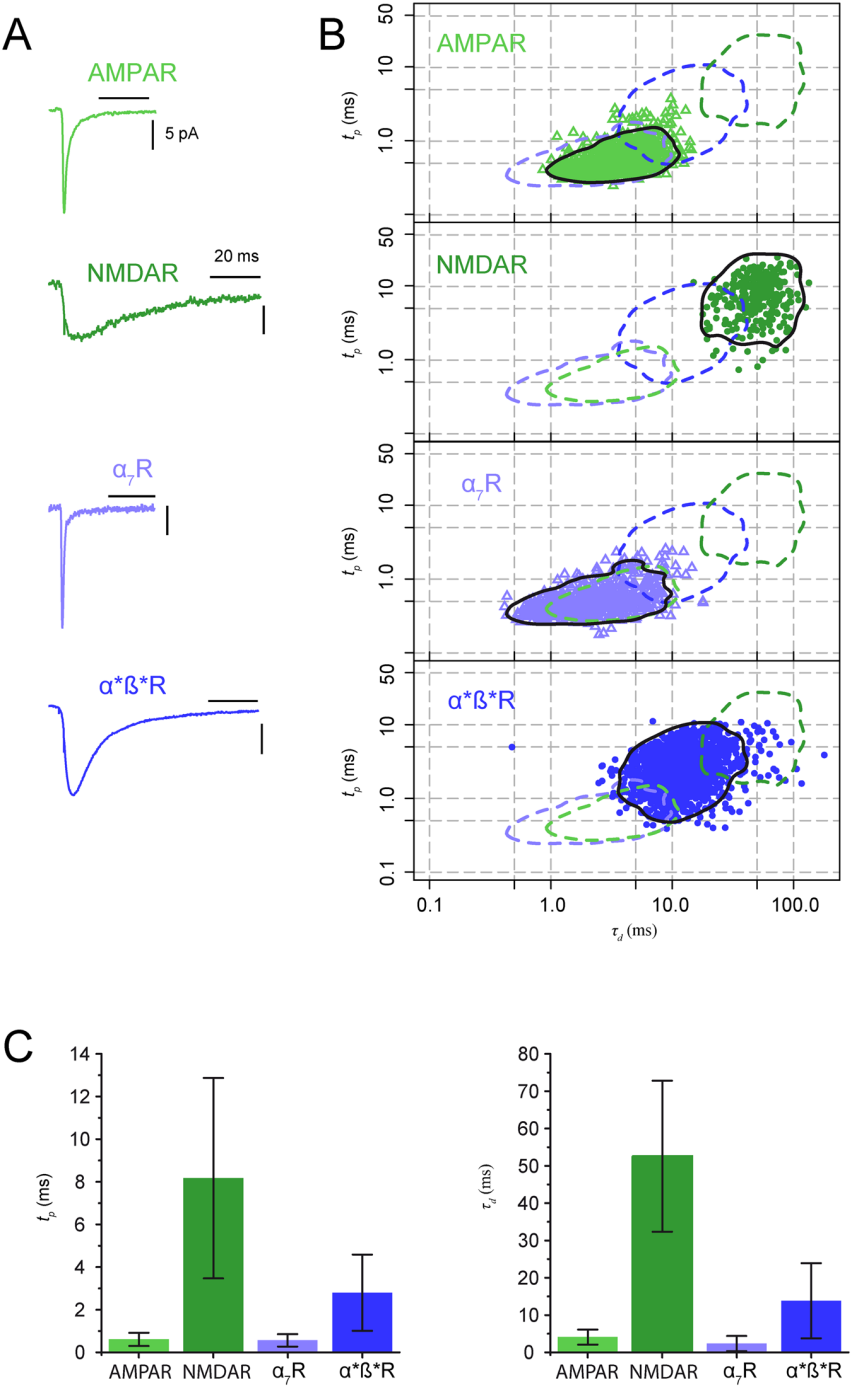
Rise time and decay of single-receptor type mEPSCS. The four types of single-receptor mEPSCs were recorded in the presence of TTX and antagonists: for AMPARs, D-APV, DHßE, MLA (N = 5 experiments, n = 1132 events); for NMDARs, NBQX, DHßE, MLA (N = 3, n = 286); for α_7_ nAChRs, D-APV, NBQX, DHßE (N = 8, n = 923); for α*β* nAChRs, D-APV, NBQX, MLA (N = 6, n = 1714). The records and the distributions illustrated were taken at −60 mV for a α_7_ and α*β* nAChRs mEPSCs, and at −40 mV for AMPAR and NMDAR mEPSCs. (**A**) Averaged records of single-receptor type mEPSCs. (**B**) *t*_*p*_ vs *τ*_d_ distributions for single-receptor type mEPSCs obtained from 5 (AMPAR), 3 (NMDAR) 8 (α_7_ nAChRs) and 6 (α*β* nAChRs) experiments. The continuous black lines define the envelope of 95% of the events, the dotted lines are the envelopes of the three other distributions. (**C**) Mean values of *t*_*p*_ and *τ*_d_ for single-receptor type mEPSCs. The two parameters were measured in individual cells and the mean and the SD were obtained from the resulting values. The fastest mean rise times (*t*_*p*_) was that of α_7_ nAChRs mEPSCs (0.51 ± 0.29 ms) and of the AMPARs mEPSCs (0.61 ± 0.31 ms). The α*β* nAChRs mEPSCs’ rise time was 2.80 ± 1.79 ms and the NMDARs mEPSCs had the slowest rise time (8.17 ± 4.69 ms). The mean decay time constants (*τ*_*d*_) of α_7_ nAChRs mEPSCs (2.38 ± 2.03 ms) and of the AMPARs mEPSCs (4.11 ± 2.02 ms) were smaller than those of α*β* nAChRs mEPSCs (13.83 ± 10.07 ms) and of NMDARs EPSCs (52.57 ± 20.23 ms).

We have measured the amplitude, rise (*t*_*p*_) and decay (*τ*_*d*_) time of mEPSCs when only one of the four receptors was available by blocking the other three with selective antagonists. Figure 2A shows the averaged mEPSCs of the four groups (each trace is from a single experiment). The mEPSCs amplitudes were measured at −60 mV for α_7_ and α*β* nAChRs and at −40 mV for NMDARs and AMPARs. The mean amplitudes (in pA) were: −24.1 ± 2.9 (n = 6 cells) for α_7_ nAChRs mEPSCs, −14.1 ± 0.9 (n = 12) for α*β* nAChRs mEPSCs, −16.1 ± 1.2 (n = 12) for AMPAR mEPSCs and −15.3 ± 2.1 (n = 9) for NMDAR mEPSCs. All these values are overestimates of the true mean values since we did not correct for missed events, a correction which would be particularly significant in the case of the NMDA mEPSCs that were detected using a high threshold because of their slow rise time.

The distribution of the values of the rise (*t*_*p*_) and decay (*τ*_*d*_) time are illustrated in Fig. 2B and C. As expected, the fastest events were those mediated by α_7_ receptors, while the slowest were those mediated by NMDARs. For each family of mEPSCs, mediated by only one of the four postsynaptic receptors, the values of *t*_*p*_ and *τ*_*d*_ showed a significant variability leading to a variable degree of overlap in the two parameter space (see scatter plot, Fig. 2B). The envelopes containing 95% of the events in the distributions of all single-receptor type mEPSCs are reported for comparison in each plot (see methods for details of the envelope calculations). The bar graphs of Fig. 2C show the mean and SD values for the rise and decay time of each of the four receptors.

### Detection of mixed unitary EPSCs

**Figure 3:**
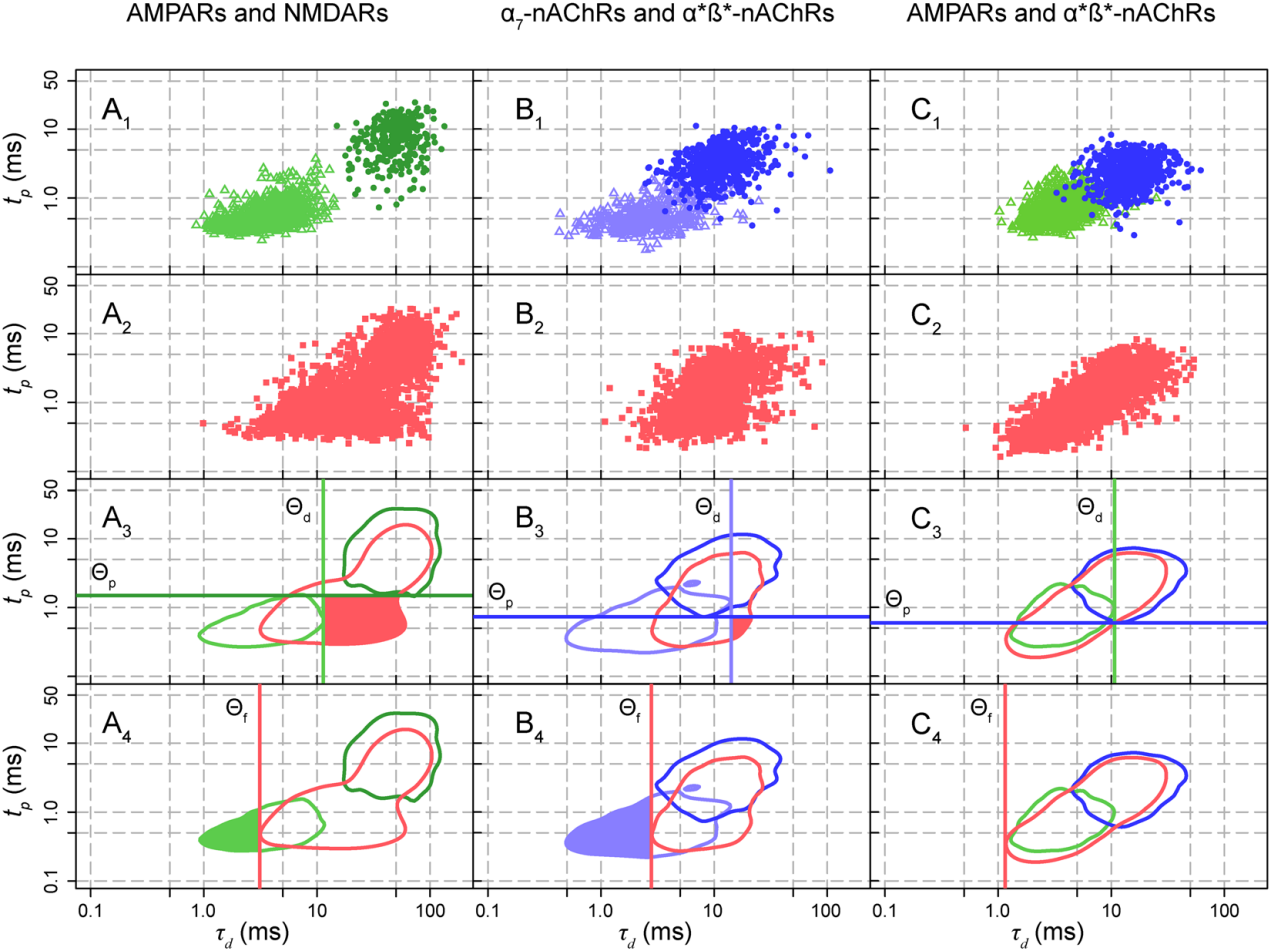
Co-localization of post-synaptic receptors deduced from pooled data. (**A**) AMPAR and NMDAR mEPSCs were collected at −40 mV in 8 experiments. In 3 experiments AMPARs were subsequently blocked with NBQX and in the others NMDARs were blocked with D-APV. **A**_**1**_. Superimposed log(*t*_*p*_) vs log(*τ*_*d*_) distributions of the two single receptor mEPSCs: 1131 AMPAR mEPSCs from 5 experiments (open triangles, light green); 285 NMDAR mEPSCs from 3 experiments (filled circles, dark green). **A**_**2**_ Distribution of mEPSCs recorded in the two-receptor situation (8 experiments). **A**_**3**_ The 95% envelopes of the distributions shown in A_1_ and A_2_ were used to define *τ*_*d*_ = θ_d_ (light green) and *t*_*p*_ = θ_p_ (dark green) and to separate four quadrants (see methods). The lower right quadrant (shaded red sector), which is nearly empty in the superimposed single receptor plot (A_1_), becomes the most populated in the two-receptor plot (A_2_), with 850 out of 2375 mEPSCs (*µ* = 35.8%). **A**_**4**_ Fast rising events appear when D-APV is bath applied to block the slow events from A_2_ to A_1_. The fraction of AMPAR events with a decay time constant faster than *θ*_*f*_ (the smallest value of *τ*_*d*_ for the envelope of the two-receptor mEPSCs) (light green sector) was 287/1131 (*φ* = *25*.*4%*.). (**B**) mEPSCs mediated by α_7_ and α*β* nAChRs were recorded at −60 mV. **B**_**1**_ Single receptor α_7_-nAChR mEPSCs (purple open triangles, 5 experiments) and α*β*-nAChR mEPSCs (blue circles, 2 experiments). **B**_**2**_ mEPSCs recorded in the two-receptor situation (red squares, 7 experiments) **B**_**3**_ 95% envelopes of the three distributions (α_7_, purple; α*β*, blue; two-receptor, red). In one receptor conditions the lower left quadrant contained 334 α_7_ events and 5 α*β* mEPSCs; the upper right quadrant 187 α*β* events and 2 α_7_ events; the lower right quadrant contained 3 α_7_ and 2 α*β* events. In two-receptor conditions 75 out of 2424 mEPSCs were found in the lower right quadrant (*µ* = 3.1%). **B**_**4**_ among the 427 α_7_-nAChR mEPSCs shown in Fig. B1, 210 (purple area in B_4_) had *τ*_*d*_ < *θ*_*f*_, where *θ*_*f*_ is the fastest *τ*_*d*_ observed in the two receptor conditions. (**C**) mEPSCs and sEPSCs (some recorded in presence of Sr^2+^) mediated by AMPARs and α*β* nAChRs were recorded at −60 mV in 10 experiments. **C**_**1**_ single receptor conditions: AMPARs (light green open triangles, 2 experiments), α*β* nAChRs (blue filled circles, 8 experiments). **C**_**2**_ mEPSCs and sEPSCs recorded in the two-receptor condition (red squares, 10 experiments). **C**_**3**_ 95% envelopes of the three distributions (AMPARs green, α*β* nAChRs blue, two-receptor red). No significant shaded area is present in the lower-right quadrant. The lower left quadrant contains 521 AMPAR events and 1 α*β*-nAChR events; the upper right quadrant contains 547 α*β* events and 11 AMPAR events. The lower right quadrant contains 2 AMPAR events and 7 α*β* events. In two-receptor conditions 16 out of 2192 mEPSCs were found in the lower right quadrant (*µ* = 0.7%). **C**_**4**_ DHßE does not induce the appearance of fast events. *φ* = *2*/*981* = *0*.*2%*.

The mEPSCs were also recorded in various conditions where two out of four receptors were available to mediate the postsynaptic current. The rise times and decay constants for the two- and one- receptor conditions were compared by superimposing their distributions in the logarithmic space. This approach is illustrated in Fig. 3 for the three main receptor pairs that we have studied: AMPARs-NMDARs (Fig. 3A), α_7_-α*β* nAChRs (Fig. 3B) and AMPARs - α*β* nAChRs (Fig. 3C). For all pairs of receptors, the presence of mixed mEPSCs was demonstrated by two features, first described in the case of AMPARs-NMDARs distributions, with nicotinic and inhibitory transmission blocked:

1. The clusters corresponding to rise and decay times of AMPARs and NMDARs mediated mEPSCs are well separated (Fig. 3A1). However, when both receptors are available (Fig. 3A2), a large proportion of mEPSC coordinates occupies the lower right quadrant, corresponding to a combination of the fast rise of AMPAR mediated mEPSCs and the slow decay of NMDAR mediated mEPSCs (Fig. 3A2 and filled red domain in Fig. 3A3). Such fast rise-slow decay events were never observed among pure AMPAR or NMDAR mEPSCs and are thus readily interpreted as “mixed” mEPSCs.
2. A second major difference between the distribution of single-receptor type mEPSCs and the distribution of two-receptor mEPSCs is the absence in the latter of a population of fast-rising, fast-decaying events present in the lower left quadrant in the single-receptor distribution (shaded area in Fig. 3A4). This absence is readily explained if in the two-receptor conditions the addition of a NMDAR component to the fast AMPAR mediated mEPSCs slows these mEPSCs and displaces them to the right in the distribution.

**Figure 4:**
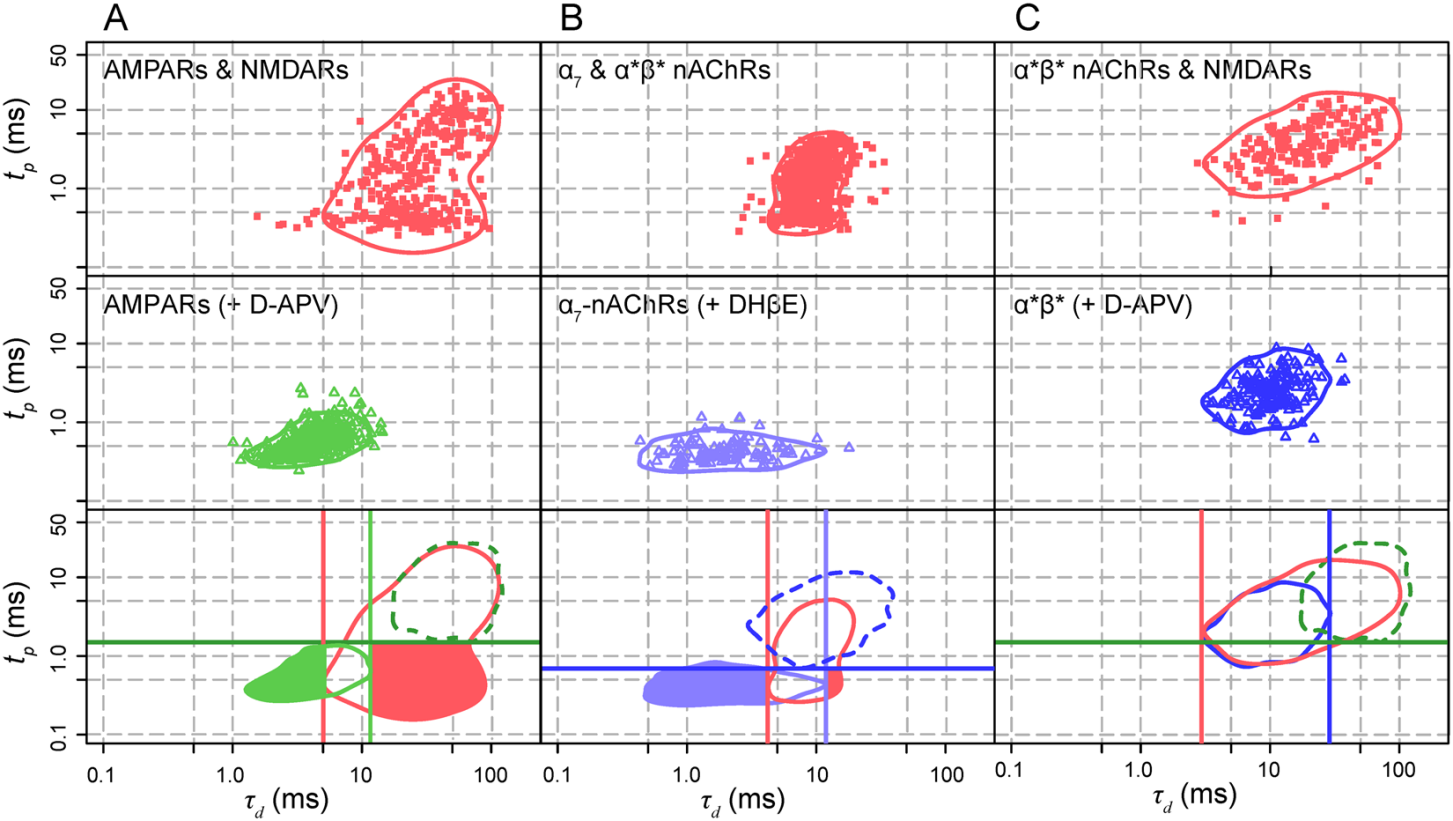
Co-localization of post-synaptic receptors deduced from single cell experiments. *Left column*. Co-localization of AMPARs and NMDARs. Glutamatergic mEPSCs were collected at −40 mV. The upper plot (red) shows the distribution of the 345 mEPSCs in two-receptor conditions. The middle plot (green) shows the distributions of the 473 mEPSCs obtained after addition of D-APV. In the lower plot the two distributions are superimposed on the envelope of the distribution (dotted line) obtained from multiple single-receptor NMDARs,experiment (green dotted line).This allows the calculation of *µ* = 149/345 = 0.43 and *φ* = 326/473 = 0.69. *Middle column*. Co-localization of α_7_ and α^*^ß^*^ nAChRs. Nicotinic mEPSCs were collected at −60 mV. Upper row (red): 585 mEPSCs recorded in the two-receptor conditions. Middle row (purple): 110 α_7_ nAChR mEPSCs recorded after addition of DHßE. Lower row: Superimposition of the two distributions obtained with the envelope of a third distribution (blue dotted line) obtained from pooled single-receptor α^*^ß^*^ nAChR experiments (Fig. 1B). This allows the calculation of *µ* = 26/585 = 4.4% and *φ* = 96/110 = 87.3%. *Right column*. Absence of mixed mEPSCs associating α^*^ß^*^ nAChRs with NMDARs. mEPSCs were collected at at −40 mV. Upper row (red): distributions of the 187 mEPSCs in two-receptor conditions (α^*^ß^*^ nAChRs and NMDARs). Middle row: distributions of the 285 mEPSCs obtained after addition of D-APV (blue). Lower row: superimposition of the distributions obtained in each of the two cells with the envelope of distributions for NMDARs mEPSCs obtained from pooled single-receptor experiments (green dotted line). This leads to the values of *µ* = 2/187 = 1.0% and *φ* = 0/160 = 0%.

These observations led us to define the two indices, *µ* and *φ*, (see Methods) to quantify respectively the proportion of events with the characteristics of the two available receptors and the proportion of events mediated mainly by the fast receptor that were partially slowed down by the second (slower) receptors. *µ* is therefore the proportion of events presenting simultaneously the rise time of the fastest receptors available and the decay due to the slowest receptors available; whereas *φ* is the proportion of mEPSCs mediated by the fast receptors that are faster than the population of events described by the 95% envelope of the mEPSCs mediated by the two populations of receptor (fast and slow). These indices were calculated by analyzing two types of distributions: a series in which pooled data from multiple experiments were combined and allowed to define the envelopes for the mEPSCs mediated by a single type of receptor and those mediated by two types of receptors (Fig. 3) and data from single cell experiments in which one could compare the distributions from two-receptor to one receptor after addition of an antagonist (Fig. 4). These two indices proved to be complementary in the detection of mixed mEPSCs associating either AMPARs and NMDARs or α_7_ and α*β* nAChRs, and both gave values close to zero in the search for mixed events associating one population of GluRs and another of nAChRs.

### Mixed AMPAR and NMDAR mEPSCs

In Fig. 3A the three distributions to be compared were obtained by combining the data from multiple experiments: 5 from AMPAR only and 3 from NMDAR only (Fig. 3A1), with the same 8 cells recorded initially in the two-receptor conditions (Fig. 3A2). Most of the experiments were performed at −40 mV to reduce the Mg block of NMDA receptors observed at the otherwise standard value of −60 mV. Figure 3A3 illustrates the presence of mixed events in the two -receptor situation. The log_10_(*τ*_*d*_) – log_10_(*t*_*p*_) space was divided into four quadrants using the two boundaries of the 95% envelopes: *θ*_*p*_ = 1.5 ms – the minimum value of *t*_*p*_ for the slow mEPSCs envelope (NMDARs) – and *θ*_*d*_ = 11.3 ms – the maximum value of *τ*_*d*_ for the fast mEPSCs envelope (AMPARs). The lower right quadrant is nearly empty in the single-receptor type situations, but in the two-receptor situation it is heavily populated with mEPSCs with a fast “AMPAR” *t*_*p*_ and a slow “NMDAR” *τ*_*d*_, i.e. mixed mEPSCs (red area in Fig. 3A3). The fraction of mixed events found in this quadrant over all the events was *µ* = 850/2375 = 35.8%.

Figure 3A4 provides a second indication of the presence of mixed events in the two-receptor situation. It illustrates that blockade of the NMDARs gives rise to the appearance of fast AMPAR-mediated events which were not present in the two-receptor situation since they were slowed by their association with a NMDAR component. To quantify this effect we defined the threshold *θ*_*f*_ = 2.6 ms: the lowest value of τ_*d*_ for the envelope of the two-receptor mEPSCs. We found that 287 out of the 1131 fast AMPAR events had a faster decay than the mEPSCs with the shortest decay time recorded in the two-receptor situation, giving a ratio *φ* = 25.4%.

### Mixed α_7_ and α*β* nAChR mEPSCs

In these experiments glutamate receptors were blocked and the mEPSCs were recorded at −60 mV, first in the “two-nAChR” conditions and then after addition of either DHßE (3 µM) or MLA (10 nM). While these experiments were conceptually similar to those described above for AMPARs and NMDARs, they were complicated by two differences. The first was the more marked overlap of the distributions of time courses of the single-receptor type events. The second was the lower frequency of the mEPSCs: in some cells the number of events collected in an hour was less than 10, and even when events were more frequent they were often pure “slow” α*β* events. Despite these difficulties, in 10 experiments it was possible to record mEPSCs both in the two-receptor situation and after blockade of either α*β* receptors (by DHßE, n = 5) or α_7_ receptors (by MLA, n = 5).

Figure 3B1–B4 illustrates the results of these experiments. The fraction of events in the lower right quadrant outside the 95% envelopes of either α*β* or α_7_ mEPSCs is *µ* = 3.1%, much smaller than in the case of Fig. 3A. On the other hand, a large fraction of the α_7_ mEPSCs recorded after addition of DHßE occupies a region in the plane which was empty in the two-receptor situation (*φ* = 49.2%), suggesting that the α_7_ mEPSCs observed in the single-receptor conditions were slower “mixed” α_7_-α*β* mEPSCs in the two-receptor conditions.

These results indicate that a single vesicle of ACh can activate the two types of nAChRs, which are therefore co-localized in at least a fraction of the synaptic contacts.

### Absence of mEPSCs or sEPSCs associating nAChRs and GluRs

Four combinations of antagonists could be used to isolate mEPSCs or sEPSCs combining specific nicotinic and a glutamatergic components: α*ß* nAChRs-AMPARs, α*ß* nAChRs-NMDARs, α_7_ nAChRs-NMDARs, and α_7_ nAChRs-AMPARs events and in each case try to reveal the association by blocking one of the receptors. We selected the first two combinations because of the low frequency of α_7_ events and considerable overlap of the distributions for AMPAR and α_7_ nAChR events. In both cases we recorded the two-receptor mEPSCs or sEPSCs and then suppressed the slow component (α*ß* in the couple α*ß*-AMPAR, NMDAR in the couple α*ß*-NMDAR) to allow the eventual detection of fast events that could only be observed in the single-receptor conditions.

### α*ß* nAChRs-AMPARs

Figure 3C illustrates the results of 10 experiments in which mEPSCs were first recorded (at −60 mV) in the presence of MLA and D-APV and later after addition of either DHßE or NBQX. There is no evidence for mixed events in the lower right quadrant where such events would be expected if a nicotinic component slowed the decay of AMPAR current. Furthermore, the addition of DHßE did not result in the appearance of a substantial number of fast events not recorded previously in the two-receptor conditions. The average values of *µ* and of *φ* were 0.8% (16/2192) and 0.2% (2/981) respectively. The figure includes both experiments using mEPSCs and experiments using sEPSCs (some of which were recorded in the presence of Sr^2+^). It could be argued that for a proportion of sEPSCs, mixed events could be due to an action potential releasing vesicles at two distinct release sites facing different receptors. However the presence of such events could only increase *µ* and *φ*. The fact that we found very low values for these two parameters with sEPSCs thus reinforces the conclusion from the experiments using mEPSCs that there is no evidence for co-localization of α*ß* nAChRs and AMPARs.

### α*ß* nAChRs-NMDARs

In a set of 9 experiments mEPSCs were first recorded (at −40 mV) in the presence of MLA and NBQX and later after addition of D-APV. Blockade of the slow NMDAR component did not eliminate events outside of the NMDAR envelope and did not result in the appearance of fast α*ß* events not evident in the two-receptor conditions. The mean value of *µ* was 0.3%, and the mean value of *φ* was 1.6%.

In the experiments combining NMDARs and α*β* nAChRs, there was often a marked asymmetry between the large numbers of NMDAR events (which arise from interneurons as well as from MNs) and the smaller numbers of α*β* nAChR events (which arise only from MNs). The presence of “pure NMDAR” events from interneurons will reduce the fraction *φ* of fast events detected after addition of D-APV. To reduce the consequences of this asymmetry we repeated the experiments at −60 mV (data not shown). At this potential most of the pure NMDAR events are too small and too slow to be detected but the slow NMDAR components of mixed events are detectable if they follow faster and larger α*β* events. This property has been used previously to demonstrate that a NMDAR component is present in mEPSCs near resting potential^11, 12^. The α*β*-NMDAR two-receptor distribution at −60 mV was not significantly affected by D-APV. In this case, it was intractable to isolate and accurately characterize the NMDA events at −60 mV because they were too small to be detected. We therefore only computed *φ* = 0.6% in the usual way and (over-)estimated *µ* as the fraction of two events recorded in the two-receptors situation that decayed more slowly that the α*β* nAChRs events (10 out of 1743 events, *µ* < 0.6%). These results suggest the absence of two-component α*ß* nAChRs -NMDAR mEPSCs.

### Measurements from single neuron experiments confirm the data from pooled experiments

**Figure 5:**
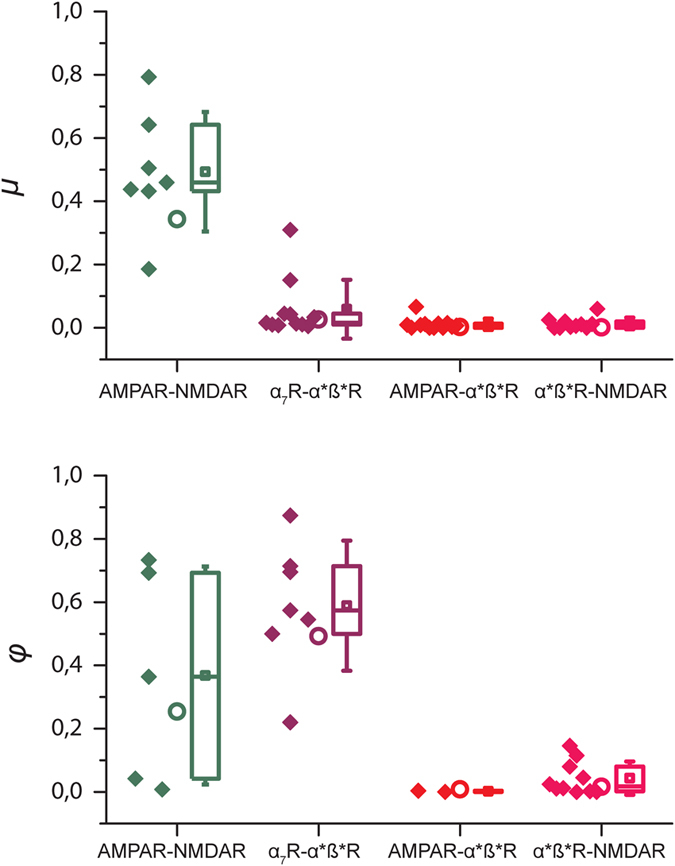
Bar graphs summarizing the values of *µ* (top) and *φ* (bottom). The upper graphs illustrate the values of *µ*, the fraction of events with a fast rise and a slow decay detected in the lower right quadrant in the initial two-receptor situation and eliminated when an appropriate antagonist blocks the slower receptor (D-APV for the NMDAR-AMPAR couple, DHßE for the α_7_-α*β* nAChR couple, NBQX for the AMPAR-α*β* nAChR couple, D-APV for the α*β* nAChR-NMDAR couple). The lower graphs illustrate the values of *φ*, the fraction of fast-rising fast-decaying events which appeared in the same set of experiments as in the upper graphs when the appropriate antagonist blocked the slower receptor. Each data set represents the value from individual experiments (symbols on the left) and a whisker plot (on the right) indicating the mean of the values for all the experiments (large symbol), the median (horizontal line) the 25 and 75 percentiles (extension of the box). The whiskers represent one standard deviation around the mean value. The circles represent the value of *µ* and *φ* obtained with the pooled events from the data illustrated in Fig. 2.

To complement the analysis of the distributions obtained by pooling results from different cells, we tried to analyze the distributions obtained from recordings from individual cells. Isolation of both single-receptor distributions from the two-receptor distribution was precluded by incomplete washout of any of the antagonists. However for a given cell, we could compare the two-receptor distribution with the single-receptor distribution following addition of an antagonist and with the envelope of the second single-receptor distribution obtained from multiple experiments. Figure 4 illustrates three such single cell experiments. The values of *µ* and *φ* derived from single cell experiments were compared with those derived from pooled experiments and the agreement was substantial, as illustrated in Fig. 5.

As shown in Fig. 5, *φ* appears as a better indicator than *μ* of the presence of mixed events since it has a high value for both AMPAR-NMDAR and α_7_-α^*^ß^*^ nAChRs, whereas *µ* is only high for AMPAR-NMDAR. This disparity is related to the difference in the kinetics of the two classes of events. In the case of *φ*, transition from the one receptor to the two-receptor conditions has a similar effect for both AMPAR-NMDAR and α^*^ß^*^- α_7_: the slowing of the fastest events by the addition of a slower component displaces the value of *τ*_*d*_ to the right and reveals the empty domain used to calculate *φ*. By contrast, in the case of *µ*, the fraction of mixed events which appear in the lower right quadrant depends on the relative kinetics of the two classes of one receptor events. In the case of AMPAR-NMDAR the rise time of the mixed event will be very similar to that of the AMPAR event because the rapid decay of the AMPA current and the slow rise of the NMDA current substantially cancel each other so that the peak of the mixed event is the same as for the AMPAR. Thus the distribution for AMPAR-NMDARs events move laterally and many of them will enter the lower right quadrant. By contrast in the case of α_7_-α^*^ß^*^ nAChRs the rise times of the two components are in the same range and the resulting rise times of the mixed events will be slower that of the AMPAR events. Hence the mixed events will move rightward but also upward and most of them will not enter the lower right quadrant, leading to a small value of *µ*.

The results represented in Fig. 5 show co-localization of a fraction of AMPARs and NMDARs, and of a fraction of α_7_ and α*ß* nAChRs. However *µ* and *φ* only give an approximate lower bound of the proportion of mixed mEPSCs, because fast events which are slowed by becoming mixed do not all move out of their region of their initial distribution.

### AMPAR-NMDAR asynchronous EPSCs

MNs are the main source of cholinergic inputs onto RCs and therefore any cholinergic mEPSC recorded from a RC is likely to originate from a MN. RCs however receive glutamatergic input from many classes of interneurons and therefore we could not ascertain from the mEPSC analysis that the co-localization of AMPARs and NMDARs applies to the synapses between MNs and RCs. This was particularly concerning since there are some doubts about the presence of vesicular glutamate transporters in MNs (see discussion) and one could envisage a possible scenario in which none of the recorded glutamatergic mEPSCs would originate from MNs.

In order to circumvent this difficulty, we examined if “asynchronous EPSCs” (aEPSCs) could be evoked by stimulation of the MN axons. aEPSCs have been shown in other systems to correspond to “uniquantal” events at synaptic terminals recently invaded by an action potential”^23^, and have been used to isolate quantal events associated with a defined set of stimulated synapses^24^. The MNs project their recurrent collaterals in lamina VII and IX of the spinal cord onto RCs and some other MNs^25–27^. Thus, by stimulating the ventral root, one elicits synaptic release from the MNs onto RCs and MNs (which in turn can also excite more RCs and yet some other MNs…). The excitatory synaptic events recorded in the RCs have therefore a single origin: the MNs, eventually through a di or polysynaptic loop. aEPSCs can be evoked by stimulation of MNs axons in the presence of external Sr^2+^ ions. We thus looked for aEPSCs in Sr^2+^ solutions and compared them with sEPSCs recorded in the same experiments and with eEPSCs elicited by “minimal stimulation” of the VRs.

The aEPSCs were collected in the 30–230 ms interval following 1p-stimulation and 5p-stimulations, and the averaged records compared to those of the eEPSCs evoked by minimal stimulation and those of the sEPSCS recorded in the same cells (and in the same Sr^2+^solution) between 2 s and 8 s after the stimulation, i.e. at a time when we could assume that delayed release had ceased. For the sEPSCs we selected experiments in which the amplitude distribution had a single peak and rejected three experiments in which the amplitude distribution of sEPSCs showed multiple peaks, since this could indicate the presence of EPSCs elicited by spontaneous action potentials. On the other hand the amplitude distribution of the eEPCSs showed multiple peaks in most cases.

**Figure 6:**
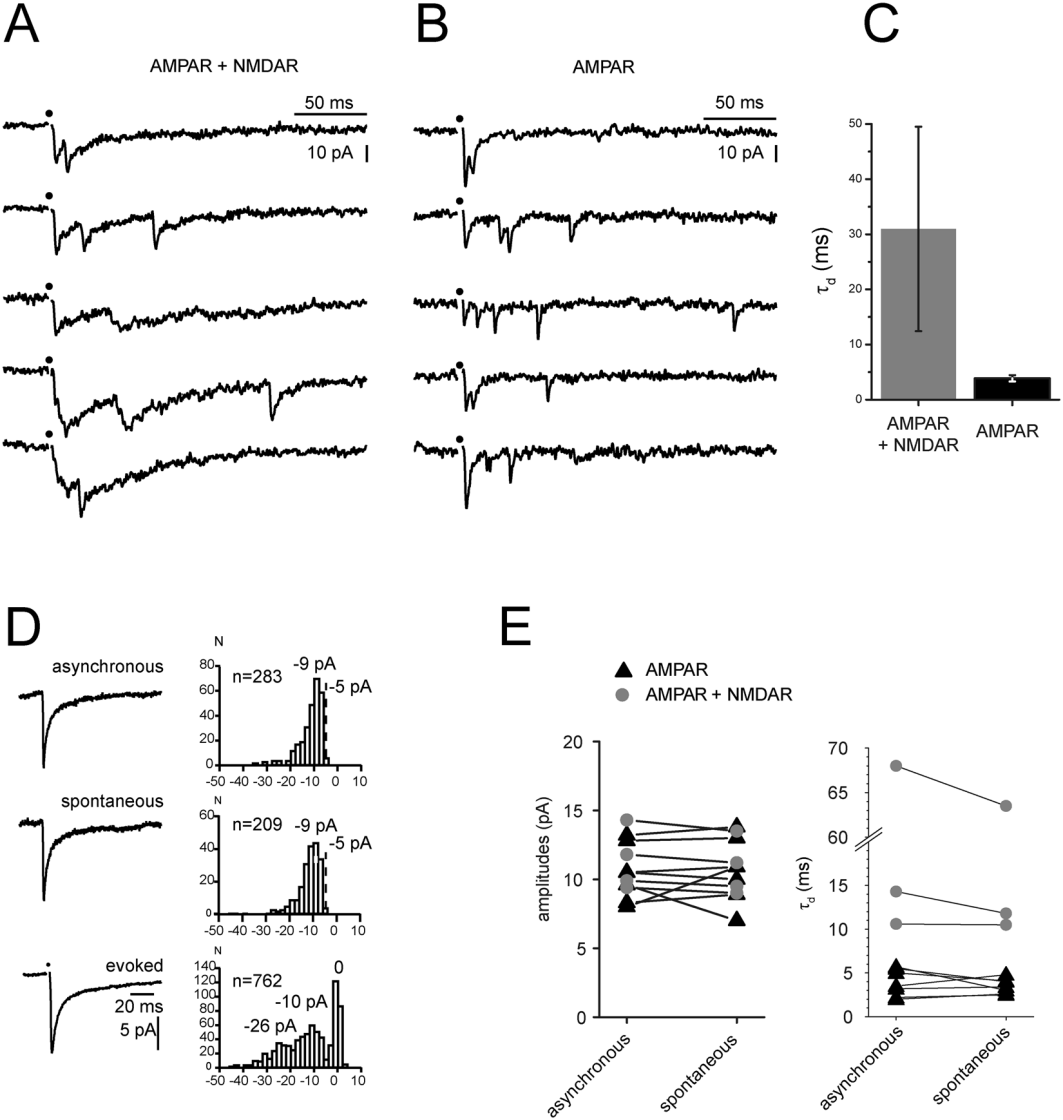
Asynchronous glutamatergic EPSCs (aEPSCs) resemble spontaneous and miniature glutamatergic EPSCs. All data were obtained at −60 mV in solutions containing Sr^2+^ (8 mM), Mg^2+^ (1 mM), no Ca^2+^ and blockers of GABA_A_ Rs, GlyRs and nAChRs. (**A**,**B**) VR stimulation with a near threshold intensity elicited an evoked EPSC (eEPSC) followed by aEPSCs. Each column illustrates five records from the same cell. In **A**, neither AMPARs nor NMDARs were blocked. (**B**) As in A, but after addition of D-APV. (**C**) Comparison of the decay time constants of the fast rising aEPSCs (with a rise time of less than 1 ms) before and after addition of D-APV. In the two-receptor condition, most of the fast rising aEPSCs had a slow decay, which was shortened after addition of D-APV. (**D**) Comparison of the aEPSCs, sEPSCs and eEPSCs evoked by minimal stimulation in the same cell. Neither AMPARs nor NMDARs were blocked. The histograms of aEPSCs and sEPSCs amplitudes have a single peak, the value of which matches that of the main peak in the eEPSC amplitude histogram. (**E**) Comparison of the amplitudes and of the decay time constants of aEPSCs and sEPSCs recorded in the absence and presence of D-APV.

Figure 6A and B illustrate records of eEPSCs and aEPSCs induced by 1p-stimulations at 0.1 Hz in Sr^2+^ before (Fig. 6A) and after (Fig. 6B) block of NMDA receptors. Blockade of NMDA receptors substantially speeds up the decay time constant (Fig. 6C). Rise time measurements from all events were faster than 1 ms, indicating that pure NMDA aEPSCs did not occur. The large difference in the decay time constants of the two groups of aEPSCs implies that in the two-receptor conditions the slower decay is due to the presence of a substantial number of mixed AMPAR-NMDAR aEPSCs. We can thus conclude that there are co-localized AMPARs and NMDARs at some of the glutamatergic MN terminals synapses.

Figure 6D shows that the amplitude distributions of aEPSCs and sEPSCs are similar in one example cell. The amplitude distributions of EPSCs evoked by minimal stimulation instead comprises multiple peaks as well as failures. The multiple peaks correspond to different numbers of vesicles released at presumably distinct release sites on any given stimulus, since a single MN axon makes a series of contacts with a given RC dendrite^28, 29^. However, within a minimal stimulation protocol, the response to a single vesicle corresponds to the distance between the peak corresponding to the failures and the first neighbouring peak of the distribution. The mean value of this “evoked quantum” for AMPAR (11.0 ± 1.9 pA) and AMPAR-NMDAR eEPSC (10.2 ± 0.5 pA) was not significantly different from the mean value of the sEPSCs recorded in the same conditions (AMPAR 9.0 ± 0.9 pA, p = 0.14 vs eEPSCs; AMPAR-NMDAR 9.5 ± 0.5 pA, p = 0.24).

Figure 6E summarizes the comparisons of the amplitude and decay time constants of aEPSCs and sEPSCs recorded at −60 mV in the presence of D-APV. The amplitudes of sEPSCs and aEPSCs are similar despite the fact that the aEPSCs originate only in MN terminals whereas sEPSCS are likely to originate from both MNs and interneurons. Decay time constants in two-receptor conditions were reduced by addition of D-APV. The presence of mixed aEPSCs in two-receptor conditions thus supports the idea that NMDARs and AMPARs are co-localized at MN to RC synapses.

### Block of the cholinergic component of evoked EPSCs

The lack of mixed glutamatergic-cholinergic mEPSCS or sEPSCs suggests that the two neurotransmitter-receptor systems are segregated at either the pre- or post-synaptic level. However, as in the case of AMPAR-NMDAR mEPSCs, the dominance of glutamatergic events originating in interneurons rather than in MNs could have masked the presence of some mixed events. The eEPSCs evoked in the RCs comprise the sum of effects of individual quanta released from each of the synaptic contacts originating from the population of MNs that synapse onto the recorded RC. Either pre-synaptic or post-synaptic segregation of glutamatergic and cholinergic synapses would result in a change in the number of functional release sites following blockade of one neurotransmitter receptor type. We thus measured responses to ventral root stimulation from RCs in different conditions of release probability before and after blockade of nAChRs by co-application of DHβE and MLA. Bayesian quantal analysis^30^ (BQA) was used to estimate the quantal parameters before and after blockade of the cholinergic component.

**Figure 7:**
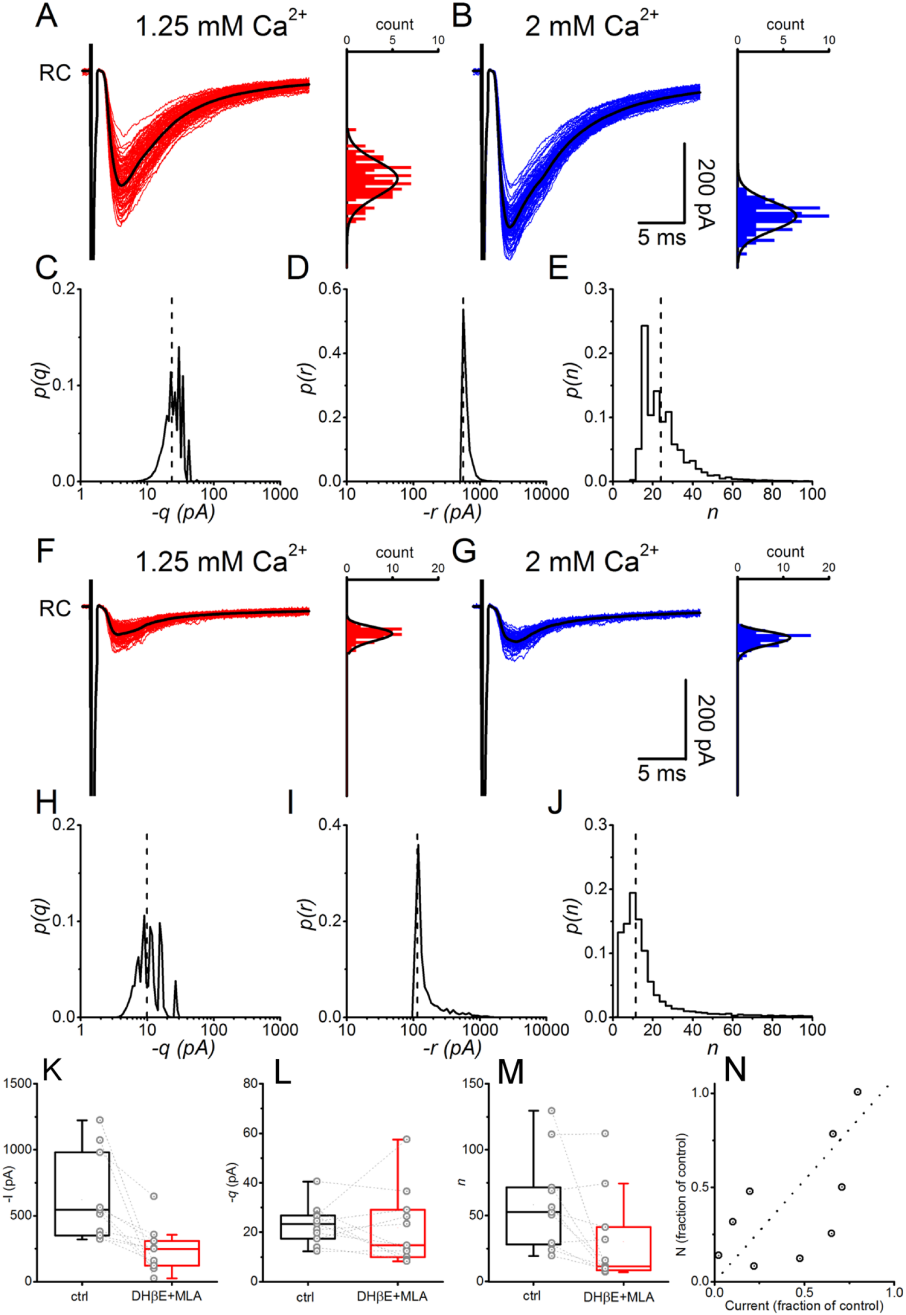
Effects of cholinergic antagonists on the quantal parameters of evoked EPSCs. Responses to VR stimulation in a RC are shown in panel A and B in two different conditions of release probability. Posterior probability distribution of the quantal parameters calculated by BQA are shown for *q* (panel C), *nq* (panel D) and *n* (panel E). The vertical lines indicate the position of the median of the distributions. The amplitude distribution histograms for each Ca^2+^ concentration are overlaid with the theoretical distributions calculated from the estimate of the quantal parameters (**A** and **B**). Following block of the cholinergic component, the evoked EPSCs were again recorded using the same two Ca^2+^ concentrations as in control. The responses are largely reduced (**F** and **G**) and the probability distributions of the quantal parameters (**H**,**I**,**J**) show that the number of release sites is reduced in the presence of the antagonist (median values indicated by a vertical line). Pooled data of the mean evoked current in control and following block of nicotinic receptors show a substantial reduction in the responses (**K**). While the difference in quantal size was not significant in the two conditions (**L**), the number of release sites was significantly reduced following block of nAChRs (**M**). The extent of change in the number of release sites is strongly linearly correlated with the fraction of the eEPSCs mediated by nAChRs (**N**).

Before block of the nicotinic component ventral root stimulation gave rise to large, Ca^2+^ sensitive, evoked currents (Fig. 7A,B), induced by synchronous antidromic spikes in MNs^29^. In this experiment, BQA determined a number of release sites of *n* = 24, with a mean quantal size of *q* = −23.4 pA. Amplitude histograms in panels A and B are overlaid with the estimated quantal amplitude distributions projected from the BQA results illustrating good overlap with the observed data. Panels C and D and E show the posterior distributions for *q*, *r* = *nq* (the maximal response) and *n* (number of release sites) obtained from the simultaneous fits of the high and low release probability datasets. The probability distribution of the number of release sites was calculated from the joint distributions of *q* and *r* ^30^. Vertical lines indicate the median of the distributions that were used as best estimates for all parameters.

Following blockade of the cholinergic component, the experimental protocol was repeated to allow comparison of quantal parameter estimates with those from the control period. Panels F and G show the reductions in evoked currents following application of the antagonists (81 and 83% in 2.0 and 1.25 mM Ca^2+^ respectively) alongside corresponding amplitude histograms and BQA-projected distributions. The quantal size and maximum current were reduced to −9.9 pA and −109 pA respectively, and the number of release sites was more than halved to *n* = 11 sites (posterior distributions are shown in Fig. 7H,I,J). In a total of 11 cells the average current was significantly reduced following cholinergic blockade from a median value of −544 pA to −248 pA (P < 0.001, Wilcoxon signed-rank test), but there was considerable variation in the effects of cholinergic antagonism across different cells (Fig. 7K). The median quantal size was −23.3 pA in control conditions and −14 pA following block of nicotinic receptors (Fig. 7L), but this difference was not statistically significant (P = 0.500, Wilcoxon signed rank test). By contrast, there was a substantial and significant reduction in the median number of release sites *n* from 53.7 to 11.5 (Fig. 7M, P = 0.014). The estimated probability of release at both Ca^2+^ concentrations did not change in the presence of the antagonist (from 0.55 to 0.60, P = 0.83 for 2 mM Ca^2+^ and 0.29 to 0.41, P = 0.11 for 1.25 mM Ca^2+^). Therefore the reduction in the size of synaptic responses results predominantly from a reduction in number of functional release sites, which confirms the segregation of the two transmitter systems.

While cholinergic blockade decreased estimates in the number of release sites, the reduction varied substantially across different cells. Since the effect of the antagonists on the average current changes in proportion to the relative contributions from the cholinergic and glutamatergic components, we compared the differences in *n* with the corresponding degree of inhibition induced by the cholinergic antagonists for each cell (Fig. 7N). As expected, in those cases in which the majority of the current was glutamatergic, the reduction in the number of release sites following blockade of the cholinergic component was minimal, while a large reduction in current corresponded to a larger reduction in the estimate of the number of release sites. The two parameters were significantly positively correlated (Pearson’s r = 0.67, P = 0.025) with a linear fit yielding a slope of 1.08. Similar analysis on the corresponding ratios of the estimated quantal sizes with the amplitudes before and during antagonist showed no significant correlation (Spearman’s r = 0.39, P = 0.24).

## Discussion

Co-localization of AMPARs and NMDARs was first observed by Bekkers and Stevens^7^ on primary cultures and subsequently confirmed in a wide variety of systems^8–12^. Our initial experiments showed this co-localization for a mixed population of AMPAR-NMDAR mEPSCs originating from both interneurons and MNs. We had to consider the possibility that the observed co-localization may not occur at MN-RC synapses, since doubts concerning the presence of VGLUTs in MNs call into question the mode of storage and release of glutamate from MNs. The presence of one or two VGLUTs in MN terminals was supported by Herzog *et al*.^31^, Landry *et al*.^32^ Nishimaru *et al*.^5^ Talpalar *et al*.^33^, but other reports have failed to show any VGLUT immunoreactivity in MNs terminals^4, 34–38^. In the absence of any VGLUT, it has been suggested that glutamate release could be carrier mediated^39^ or occur through channels^40^. These uncertainties about the mode of glutamate release from MNs led us to compare glutamatergic asynchronous EPSCs (originating from MNs) and spontaneous EPSCs (originating from both MNs and interneurons). The similarities of the two types of events suggest that at MN-RC synapses AMPARs and NMDARs are co-localized and activated by “classical” vesicular release.

The presence of mixed EPSCs comprising α_7_ and α^*^ß^*^ nAChR components has been described already in various areas of the peripheral CNS, including the chick ciliary ganglion^13, 14, 41^ and the rat submandibular ganglion^42^. On the other hand, biphasic nicotinic mEPSCs have not been described before in the mammalian CNS, even though there is evidence of mixed evoked nicotinic EPSCs in mouse cortical interneurons^43, 44^ and in pyramidal hippocampal neurons^45^. Our evidence of mixed kinetics nicotinic mEPSCs shows that at least two types of nAChRs are co-localized at the postsynaptic membrane of RCs, in contrast with the apparent segregation of α_7_* and α_4_*ß_2_* nAChRs in the substantia nigra dopaminergic neurons^46^ and of at least three types of nAChRs in the goldfish Mauthner cells^47^.

The absence of mEPSCs or sEPSCs associating GluRs and nAChRs, together with the results of the quantal analysis, establish that glutamatergic and cholinergic synapses are segregated.

Our observations contrast with those previously made on ACh - glutamate synapses described in Xenopus embryos and tadpoles, in which mixed miniature events were detected on the post-synaptic cell^20, 21^. The discrepancy suggests that in the course of development there is a segregation of the elementary structures responsible for the release of transmitters and/or their post-synaptic receptors.

This segregation could be a transient stage in a process leading to the selection of the final “single neurotransmitter” phenotype which in other systems occurs late in development^48^ and often after an initial stage in which multiple transmitters are present in the same cell^22, 49, 50^. The observation that, in the adult rat or cat, MN terminals onto RC dendrites never appose postsynaptic AMPA receptors^38^ could indicate that the glutamatergic component of the MN-RC synapse is only transient. However, our data obtained in adult mice indicate that the mixed cholinergic-glutamatergic signaling from MN to RC is preserved throughout development from young juvenile (P5-P10) to fully mature (up to 3 months) mice. This is in agreement with recent studies showing that co-transmission and co-release of glutamate and ACh can be observed in juveniles or adults: 22–28 days^51^, 6–8 weeks or adults^52^, 4–6 weeks^53^.

In principle, the segregation of glutamatergic and nicotinic synapses could be post-synaptic, pre-synaptic or both pre- and post-synaptic.

A purely post-synaptic segregation would imply pre-synaptic sites with vesicles releasing both glutamate and ACh on post-synaptic densities containing either nicotinic or glutamate receptors. ACh and glutamate can be stored and released from common vesicles, as shown in various systems by the observation of mixed mEPSCs^20, 21^ and by biochemical, immunohistochemical and genetic observations indicating that synaptic vesicles from the striatum^51, 53–56^, the interpeduncular nucleus^52, 57^ and the Torpedo electroplaque^58^ contain both the vesicular ACh transporter (VAChT) and a vesicular glutamate transporter (VGLUT3 in the striatum, VGLUT1 in the interpeduncular nucleus, VGLUT1 and VGLUT2 in the Torpedo electroplaque). In all these examples there is no evidence of a post-synaptic segregation of receptors. However, in the GABA-glycine case, in which co-release is well established in neonatal rats and mice by the presence of mixed miniature PSCs^15, 17, 18, 59, 60^ and by the fact that the vesicular transporter, VIATT, is capable of accumulating both GABA and glycine^61, 62^, there are indications that at some synapses the two post-synaptic receptors are segregated^63, 64^.

Pre-synaptic segregation would suggest separate release sites for ACh and glutamate. These release sites could face a mixed population of receptors in which only those binding the presynaptic transmitter would be activated, while the others remain “silent”. There are examples in which receptors can cluster under a “non-matched” terminal, such as glycine receptors apposing cholinergic^65^ or GABAergic terminals^66^, or muscarinic M2 receptors apposing glutamatergic boutons in MNs^67^.

Finally it is conceivable that pre-synaptic and post-synaptic segregation co-exist, with glutamate release sites facing glutamate receptors and ACh release sites facing nAChRs. In an extreme case the various types of release sites could be located in different axon arborisations. There are cases of co-transmission in which two transmitters appear to be stored in distinct vesicles; e.g., glutamate and GABA^68^, glutamate and dopamine^69^, GABA and ACh^70^. Simultaneous pre- and post-synaptic segregation has been observed also in invertebrate^71^ and vertebrate^72^ synapses.

In the case of the MN-RC synapse pre-synaptic segregation is neither supported by the finding that the vast majority (90%) of retrogradely labelled MN axon varicosities are immunoreactive for VAChT^4^, nor by the presence of both glutamate and aspartate in most of VAChT positive labelled varicosities^73^. Furthermore the presence of mixed vesicles is easily reconciled with the mixed mEPSCs seen in Xenopus embryos and tadpoles^20, 21^, and with the recent observations showing that VAChT and a vesicular VGLUT can be found in the same vesicles^51, 52, 54, 55, 58^. We thus tentatively favor the hypothesis of a post-synaptic segregation with its full demonstration pending identification of the pre-synaptic mode of glutamate storage.

## Methods

In Paris, all experiments were carried out in in accordance with the relevant French guidelines and regulations (authorization CEEA34.BLDI.068.12 issued by the Paris Descartes University Ethical Committee). In London, all experiments were in accordance with the Animal (Scientific Procedures) Act (Home Office, U.K.) 1986 with the approval of the UCL Ethical Committee, under project license number 70/7621 granted by the Home Office.

### Animals and slice preparation

C57BL/6 J mice (Janvier) (P5-P10) were anesthetized with an i.p. injection of 0.1 ml of pentobarbital (25 mM). The dissection and the slicing were performed as described previously^74^. Slices were transferred into artificial cerebrospinal fluid (ACSF) containing (in mM): NaCl 130, KCl 2.5, CaCl_2_ 2, MgCl_2_ 1, NaH_2_PO_4_ 1.0, NaHCO_3_ 26, glucose 25, Na-ascorbate 0.4, Na-pyruvate 2, bubbled with 95% O_2_ and 5% CO_2_ (pH 7.4). After a 30-minute incubation in ACSF at 34 °C, slices were maintained at room temperature (18–24 °C). Recordings from Renshaw cells in adult spinal cord slices have never been reported. However, adult motoneurons recordings have been performed by Mitra and Brownstone^75^ and more recently by Hadzipacic *et al*.^76^, while recordings from viable interneurons were obtained by Husch *et al*.^77^. Spinal cord tissue is very sensitive to anoxia, therefore, we have tried to minimize the time between the sacrifice of the animal and the slicing of the tissue. We routinely managed to cut the first spinal cord slice well within ten minutes after decapitation and this invariably led to healthy tissue and viable Renshaw cells and motoneurons. Prolonging this time resulted in deterioration of the tissue and poor cell quality. For adult slices, we maintained the composition of aACSF and slicing solution as for juvenile tissue.

### Electrophysiology

A HEKA EPC-9 or Molecular Devices Axopatch 200B amplifiers were used for data acquisition. Whole-cell recordings were filtered at 3 kHz and digitized at 10 kHz. Series resistance (range 8–40 Mohms) was corrected (60–80%) in most recordings of mEPSCs and in all the recordings of EPSCs evoked by ventral root stimulation.

The recording chamber was continuously perfused with ACSF at a rate of about 1 ml/minute. RCs were first identified by their characteristic response to ventral root (VR) stimulation and voltage-clamped in the whole-cell configuration. Experiments involving quantal analysis and those on adult animals were performed on GlyT2-EGFP mice^78^ where EGFP expression facilitated targeting cells in the Renshaw cell area. Patch pipettes had an initial open-tip resistance of 3.5 to 6 MOhms. The internal solution contained (in mM): Cs-gluconate 125, spermine 10, QX-314 Cl 5, HEPES 10, EGTA or BAPTA 10, CaCl_2_ 1, Mg-ATP 4, Na_2_GTP 0.4. The pH was adjusted to 7.3 with CsOH, and the osmolarity to 285–295 mOsm. Membrane potentials were corrected for the liquid junction potential (V_j_ = −15 mV). Except when indicated otherwise, the recordings illustrated were obtained at −60 mV.

When required, the glutamatergic components of the EPSCs were suppressed by adding 2,3-dioxo-6-nitro-1,2,3,4-tetrahydrobenzo(f)quinoxaline-7-sulphonamide (NBQX, 2 µM) to block AMPARs and D(−)-2-Amino-5-phosphonopentanoic acid (D-APV, 50 µM) to block NMDARs. In a few experiments blockade of the NMDARs was reinforced by the addition of a glycine site blocker, di-chloro-kynurenic acid (dCK, 20 µM)^79^. For nicotinic EPSCs methyl-lyc-aconitine (MLA, 10 nM) was used to block the α_7_ nAChRs, and DHßE (3 µM) to block α^*^ß^*^ nAChRs. This concentration of DHßE does not block completely the ß_4_ component of the nicotinic EPSCs^80^ but the amplitude of the mEPSCs was reduced below the detection threshold. In some experiments dual block of the nAChRs was achieved with mecamylamine (MEC, 50 µM). The contribution of GABAergic and glycinergic inputs was usually negligible at −60 mV since this potential is close to their reversal potential in our experimental conditions. We nevertheless added strychnine (1 µM) and SR 95531 (gabazine, 3 µM) in most experiments, except in the case in which we analyzed responses involving the α_7_ nAChRs where strychnine was omitted or added at 0.1 µM because at 1 µM it blocks α_7_ nAChRs^46, 81^. When strychnine was added at 0.1 µM it was verified that glycinergic mIPSCS were outward and did not contaminate the recording of nicotinic mEPSCs.

NBQX, D-APV and gabazine were purchased from Tocris and Ascent. QX-314 chloride was from Alomone Labs. All the other chemicals were from Sigma.

### Analysis of EPSCs

We analyzed four types of synaptic events: miniature EPSCs (mEPSCs), spontaneous EPSCs (sEPSCs), evoked EPSCs (eEPSCs), and asynchronous EPSCs (aEPSCs).

*Miniature EPSCs* (*mEPSCs*) were recorded in the presence of tetrodotoxin (TTX, 0.2 µM). The frequency of the nicotinic mEPSCs was low, usually on the order of one per minute or even less, but often increased after an hour or two. The frequency of glutamatergic mEPSCs was higher than that of nicotinic mEPSCs, which is likely due to the fact that, whereas MNs are the sole source of cholinergic events in RCs^82^ both glutamatergic interneurons and MNs contribute glutamatergic mEPSCs.

*Spontaneous EPSCs* (*sEPSCs*) were compared to mEPSCs. The amplitude distribution of sEPSCs sometimes showed multiple peaks, which could indicate either that synaptic sites were heterogeneous, or that some sEPSCs resulted from synaptic release by spontaneous action potentials. In conditions in which the sEPSCs amplitude distribution showed a single peak, and a single-receptor type was unblocked, both the rise time and the decay of sEPSCs were indistinguishable from those of the mEPSCs. When we looked for “mixed” events in two-receptor situations, the sEPSCs had to be analyzed with the caveat that one could not exclude the possibility that two quanta were released by different terminals from a single axon. We thus restricted the detection of mixed sEPSCs to only one of the two-receptor situations (α^*^ß^*^ nAChRs - AMPARs, Fig. 3C) in which the analysis showed no evidence for mixed events. The fact that in this case we did not observe any mixed sEPSCs reinforced our conclusions on the segregation of α^*^ß^*^ nAChRs from AMPARs.

*Evoked EPSCs* (*eEPSCs*) were elicited by stimulation of the VR. The decay of large eEPSCs can be prolonged by the electrical coupling between RCs, since the VR stimulation often induces EPSPs in the RCs electrically coupled to the cell under study, and these EPSPs will appear as slow inward synaptic currents in the cell under study^74^. However this problem was not encountered with EPSCs of low quantal content recorded in Sr^2+^ solutions (Fig. 6), since in this case eEPSCs in neighboring cells were likely of comparable size to sEPSCs and mEPSCs, and after being filtered by the electrical connection were undetectable in the recorded cell.

*Asynchronous EPSCs* (*aEPSCs*) *released after an eEPSC* ^23^, were rare in normal conditions. To increase their frequency of occurrence we used an external solution in which the Mg^2+^ concentration was kept at 1 mM but Ca^2+^ was removed and Sr^2+^ was added at a concentration of 8 mM. We analyzed the aEPSCs in the time interval between 30 and 230 ms after a single stimulation or after a train of five stimulations of the ventral root (respectively 1p- and 5p-stimulation). This study was limited to glutamatergic aEPSCs and aimed at examining if the aEPSCs originating from MNs were similar to the mEPSCs, most of which originate from interneurons.

During long series of stimulations of the VR, the AMPA component of the eEPSCs tended to reduce with time and recovered very slowly if the stimulation was interrupted. A similar “silencing” of the AMPA EPSCs has been observed in neonatal hippocampus by Xiao *et al*.^83^ who found that it could be prevented by reducing the external Ca^2+^ and by using a fast intracellular Ca^2+^ buffer like BAPTA. Indeed we found that the progressive decrease of the AMPA eEPSCs was greatly reduced by using low extracellular Ca^2+^ and by replacing our usual intracellular Ca^2+^ buffer (EGTA 10 mM) by an equimolar amount of BAPTA. Thus, in 8 of the 15 experiments on eEPSCs and aEPSCs in Sr^2+^ (Fig. 3), we used BAPTA as the intracellular buffer.

mEPSCs, sEPSCs and aEPSCs were analyzed with Neuromatic (Thinkrandom.com). The detection threshold was set between −5 and −12 pA depending on the background noise level but also on the purpose of the measurement. In the experiments in which we tried to correlate the amplitudes of sEPSCs and aEPSCs with eEPSC amplitudes (Fig. 6), we lowered the detection threshold as much as possible to reduce the overestimation of the mean value resulting from the neglect of missed events. On the other hand, in the experiments in which our primary objective was to evaluate EPSC kinetics, we raised the detection threshold to values between −7 and −12 pA to avoid the uncertainties associated with fitting small events with exponentials. This may have resulted in a slight overestimate of the mean amplitude, due to the absence of correction for the missed small events.

The rise time of the response, *t*_*p*_, was measured as the interval between 20% and 80% of the peak response. The decay time constant *τ*_*d*_ was measured by a single exponential fit. Even though the mixed mEPSCs decay comprised dual components with distinct time constants, the signal to noise ratio was too low and the time constants not sufficiently different to allow a good unconstrained double exponential fit of all single events. For both the measurement of the rise time and decay time constants we used a binomial filter with up to *Np* = 10 points whose kernel size was adjusted to the noise-signal ratio of each recording. This post-hoc filter had a cut-off frequency comprised between 2.5 kHz (*Np* = 2) down to 1.2 kHz (*Np* = 10). As a result the distributions presented in Figs 2, 3 and 4 exhibit a lower bound of around 0.4 ms on both axes. This filtering imposes a limit on the biophysical analysis of the fastest events, but it does not affect our comparison of the various distributions.

### The characterization of “mixed” mEPSCs, sEPSCs and aEPSCs

We based most of our analyses on the comparison of the rise and the decay of the unitary PSCs. The scatter distributions of rise time (*t*_*p*_) vs decay time constant (*τ*_*d*_) were plotted using logarithmic axes. The distribution densities were first calculated on a 100 × 100 grid on the log_10_(*τ*_*d*_)-log_10_(*t*_*p*_) space encompassing the whole range of the data. The density distribution was then smoothed with a gaussian kernel scaled to 1.5 times the bandwidth of the data in each dimension (equation 5.5 p127 of Venables and Ripley^84^). The density map was integrated and the density level corresponding to 95% of the events was identified. The density map was then thresholded to this level to draw the envelope of the distribution containing 95% of the events.

Figure 2 illustrates the results for mEPSCs in single-receptor experiments using data from 3 to 8 experiments. The regions containing 95% of the events were subsequently used as references for each population of mEPSCs. The distributions showed varying degrees of overlap. The regions describing α_7_ nAChR and AMPAR mEPSCs were almost completely overlapping, whereas those describing α_7_ nAChR (or AMPAR) and NMDAR mEPSCs were nearly totally separated (Fig. 2). The amplitudes of the four classes of mEPSCs were in the same range (data not shown).

To identify mixed events we compared distribution densities in two-receptor conditions with those obtained in the single-receptor conditions. We looked for two of the most conspicuous differences: the presence in two-receptor conditions of events occupying regions of the plane in which there were no events in the single-receptor conditions, and, alternatively, the disappearance in two receptor conditions of events present in the single-receptor conditions.

This led us to define two indices of the presence of “mixed” mEPSCs: the fraction of “mixed” events only observed in the two-receptor distribution the fraction of fast events only observed in the single-receptor situation.

### Quantification of the fraction (*µ*) of “mixed” events only observed in the two-receptor distribution

To evaluate the fraction of “mixed” events, we estimated how many minis shared the characteristics of the events mediated by the fast and the slow receptors. To this end, we counted the events which are present in the two-receptor distributions and absent in the single-receptor distributions. We divided the log_10_(*t*_*p*_)-log_10_(*τ*_*d*_) space into four quadrants (fast and slow rise times, fast and slow decay time constant). The envelopes containing 95% of the events in the “one receptor conditions” were used to determine the boundaries (*θ*_*p*_ and *θ*_*d*_) of these quadrants. *θ*_*p*_ is the rightmost border of the distribution envelope containing 95% of the fast events (i.e. the slowest decay time constant of the events mediated by the fast receptors), and *θ*_*d*_ is the lower border of the distribution envelope containing 95% of the slower events (i.e. the fastest rise time observed among the events mediated by the slow receptor). We then counted the number of events present in the lower right quadrant (which encompass events with a fast rise and a slow decay). Due to the definition of *θ*_*p*_ and *θ*_*d*_, from which the lower right quadrant is defined, these fast rising-slowly decaying events were not observed when only one receptor was available to mediate the synaptic current.

The value of *µ* is an underestimate of the true fraction of mixed mEPSCs because some mixed events are likely to be present in the quadrants of the fast (down-left) and slow (up-right) mEPSCs when only one receptor is available, but might go undetected if the relative contributions from each component are too different. We did not try to correct for this underestimation and only used the values of *µ* as an approximate lower bound of the proportion of mixed mEPSCs.

### Quantification of the fraction (*φ*) of fast events only observed in the single-receptor situation

The rise and the decay of mixed mEPSCs originating from the combination of fast and slow mEPSCs are on average slower than those of the fast mEPSCs and faster than those of the slow minis. Thus most of the mixed events “condense” in a central region overlapping the domains occupied by the fast and the slow mEPSCs. Conversly, the addition of an antagonist blocking the slow receptors is expected to unmask the fastest mEPSCs from a population of mixed events. These fast events will appear at the left of the distribution of mixed mEPSCs. In order to evaluate this effect, we defined a third threshold *θ*_*f*_ the minimal value of *τ*_*d*_ in the envelope of the mEPSCs recorded in presence of two-receptors. The fast-decaying (and fast-rising) events uniquely observed in the “one-receptor” situation are found on the left of this line (filled areas in Fig. 3A4,B4). Their proportion (with respect to the fast events in the one receptor condition) – *φ* – was used to evaluate the presence of mixed events. In the example of Fig. 3A4, θf = 2.61 ms and among the 1131 events recorded when only the AMPA receptors (fast) mediated the miniature postsynaptic currents, 287 had a decay time smaller than *θ*_*f*_ (red in Fig. 3A4), giving *φ* = 25.4%.

### Statistical analysis

For mEPSC, sEPSC, and aEPSC recordings, Student’s t-tests (paired unless otherwise stated) were used to assess the difference between two samples. The level of significance used was p < 0.05, and the figures were labeled according to the value of p (*p < 0.05; **p < 0.01; ***p < 0.001). Summary results are expressed as mean ± SEM except in Fig. 1 where SD was used. Since the results of quantal analysis were not normally distributed, non-parametric Wilcoxon sign rank tests were used to test for significant differences in the quantal parameter estimates across the group data.

### Bayesian quantal analysis

Bayesian quantal analysis (BQA) was performed to estimate the quantal parameters using probabilistic modelling^30^. Whereas multiple-probability fluctuation analysis^85^ is based on fitting amplitude variances to mean amplitudes at different probabilities of release, BQA models simultaneously the profile of every amplitude distributions at all observed probabilities using a likelihood function that combines a binomial model of release and gamma probability density function for the distribution of uniquantal events amplitudes. Within this framework, the number of release sites *n*, the probability of release *p* and the quantal size *q* are estimated from the data. The technique confers the advantage of yielding reliable estimates of the quantal parameters from small data sets^30^.
